# Incidental Parathyroidectomy during Total Thyroidectomy: Risk Factors and Consequences

**DOI:** 10.1155/2016/7825305

**Published:** 2016-08-18

**Authors:** Dimitrios K. Manatakis, Dimitrios Balalis, Vasiliki N. Soulou, Dimitrios P. Korkolis, Georgios Plataniotis, Emmanouil Gontikakis

**Affiliations:** Department of Surgical Oncology, Agios Savvas Anticancer Hospital, 11522 Athens, Greece

## Abstract

*Objective*. To evaluate the incidence of accidental parathyroidectomy in our series of total thyroidectomies, to investigate its clinical and biochemical consequences, and to identify potential risk factors.* Methods*. Patients who underwent total thyroidectomy between January 2006 and December 2015 were retrospectively analyzed. Pathology reports were reviewed to identify those cases who had an incidental parathyroidectomy and these were compared to patients with no parathyroidectomy, in terms of clinical (age, sex, and symptoms of hypocalcemia), pathological (thyroid specimen weight, Hashimoto thyroiditis, and malignancy), and biochemical (serum calcium and phosphate levels) factors.* Results*. 281 patients underwent total thyroidectomy during the study period. Incidental parathyroidectomy was noticed in 24.9% of cases, with 44.3% of parathyroid glands found in an intrathyroidal location. Evidence of postoperative biochemical hypocalcemia was noticed in 28.6% of patients with parathyroidectomy, compared with 13.3% in the no-parathyroidectomy group (*p* = 0.003). Symptomatic hypocalcemia was observed in 5.7% and 3.8%, respectively (*p* = 0.49). Age, sex, thyroid specimen weight, Hashimoto thyroiditis, and malignancy did not differ significantly between the two groups. *Conclusions*. Our study found an association of incidental parathyroidectomy with transient postoperative biochemical hypocalcemia, but not with clinically symptomatic disease. Age, sex, thyroid gland weight, Hashimoto thyroiditis, and malignancy were not identified as risk factors.

## 1. Introduction

Standardization of the thyroidectomy technique and advances in perioperative management have led to significant decrease in the overall mortality and morbidity over the past decades. Postoperative hypocalcemia remains the single most common complication of thyroid surgery, with a prevalence ranging between 16 and 55%, depending on the definition of low calcium levels [1–3]. Most cases are transient and asymptomatic, documented only biochemically; however permanent hypocalcemia can be debilitating for the patient, causing life-long dependency on oral calcium supplementation, significant loss of quality of life, and medicolegal issues against surgeons.

Acute parathyroid insufficiency is considered as the main factor affecting postoperative calcium levels and occurs due to incidental parathyroidectomy (IPT), parathyroid gland (PG) injury, or devascularization [3].

With an incidence of 3–30%, IPT is a relatively common finding in thyroid pathology reports, even in the hands of experienced endocrine surgeons [2, 4]. There is no consensus however regarding predisposing factors and high-risk patients, as well as its biochemical and clinical consequences. The aim of this study was to determine the incidence of IPT in our series, to evaluate its impact on postoperative calcium levels and to identify potential risk factors.

## 2. Materials and Methods

After institutional ethics committee approval, a retrospective analysis was undertaken, of prospectively collected data, between January 2006 and December 2015, at the Department of Surgical Oncology, Agios Savvas Anticancer Hospital, Athens, Greece. Included were all consecutive patients, aged ≥18 years, with no prior history of neck exploration or irradiation, scheduled to undergo total thyroidectomy for benign or malignant pathology on preoperative FNA biopsy. Excluded were patients who underwent lobectomy ± isthmectomy, subtotal thyroidectomy, cervical lymph node dissection or reoperation procedures, patients with coexisting parathyroid pathology, chronic renal impairment and other conditions affecting calcium metabolism, and patients on oral calcium supplementation.

All operations were performed by two experienced surgeons or by residents under their direct supervision, with careful dissection along the thyroid capsule, using an electrothermal bipolar vessel sealing system (LigaSure Precise or LigaSure Small Jaw, Covidien, USA) and no suture ligations. Intraoperative visualization of the recurrent laryngeal nerves and PGs was routinely attempted, but excessive dissection to search for missing PGs was avoided. After macroscopic examination, if a PG was identified within the surgical specimen, it was placed in iced saline until the end of the procedure. Following slicing into 1 × 1 mm pieces, those were implanted into separate muscle pockets in the sternocleidomastoid muscles, closed by nonabsorbable Prolene 4/0 sutures. The thyroidectomy specimens were wholly embedded in paraffin cubes and serial gross sections every 2 mm were obtained for histopathology.

Serum calcium was adjusted to preoperative albumin levels. Biochemical hypocalcemia was defined as total serum calcium concentration <8 mg/dL (institutional reference range 8.0–10.3 mg/dL). Clinical hypocalcemia included signs and symptoms of tetany, muscle cramps, and paresthesia. Patients with permanent hypocalcemia were still on treatment more than 6 months after surgery, whereas transient hypocalcemia was defined as correction of falling serum calcium levels within 6 months postoperatively. Hyperphosphatemia was defined as serum phosphate concentration >4.5 mg/dL (institutional reference range 2.7–4.5 mg/dL). Serum calcium and phosphate levels were measured at 12 and 24 hours postoperatively and uncomplicated cases were discharged on the first postoperative day. Symptomatic patients and patients with evidence of biochemical hypocalcemia were initiated on an oral supplementation regime of 1000 mg calcium carbonate/800 IU vitamin D3 three times daily and were discharged from hospital when symptoms resolved and calcium levels were restored above 8 mg/dL. Hypocalcemic patients were followed up in the outpatient department at weekly intervals, with serial calcium and phosphate measurements, with gradual step-down of the supplementation dosage.

Demographic characteristics, operative findings, and biochemical test results were documented for all patients. Pathology reports were reviewed independently by two authors to determine the incidence of IPT, the number and size of PGs removed, and their location in conjunction with the thyroid capsule (extracapsular, subcapsular, and intrathyroidal), as well as final diagnosis and thyroid gland weight.

On the basis of parathyroidectomy occurrence, patients were divided into two groups (incidental parathyroidectomy, IPT group, and nonincidental parathyroidectomy, non-IPT group). Those groups were compared in terms of age, sex, thyroid gland weight, presence of thyroiditis, and malignancy in the final pathology report, as well as postoperative serum calcium levels and clinical, transient, and permanent hypocalcemia rates. To further assess the impact of the number of resected PGs, pre- and postoperative serum calcium and phosphate levels were compared between three subgroups (group A: no IPT, group B: one PG resected, and group C: two or more PGs resected).

Statistical analysis was performed on SPSS version 20.0, using* t*-test and ANOVA for numeric variables and chi-square for categorical variables. Statistical significance was set to *p* < 0.05.

## 3. Results

In the course of the study period, a total of 281 patients (63 males, 218 females) underwent total thyroidectomy in our department (Table 1). Histopathologic examination of the resected specimen revealed benign disease and malignancy in 195 (69.4%) and 86 (30.6%) cases, respectively. Parathyroid tissue was verified histopathologically in 70 (24.9%) cases (Table 2).

In most patients with IPT (48/70, 68.6%), only one PG was removed. However, 14 (20%) and 3 (4.3%) patients had two or three PGs removed, respectively. In 5 cases (7.1%), only a fragment of parathyroid tissue was identified, after thorough examination. Mean diameter of resected PGs was 4.1 mm.

In 44.3% (31/70) of cases, the PGs were completely intrathyroidal, surrounded by thyroid parenchyma. In another 28 cases (40%) they were found in a subcapsular position, while 11 (15.7%) were located outside the thyroid capsule. The vast majority of extirpated PGs was normal (66/70, 94.3%); however in 4 patients they showed mild hyperplasia without atypia. No parathyroid adenomas were discovered.

Transient biochemical hypocalcemia was evident in 48 (17.1%) patients; however only 12 (4.3%) patients presented with clinical symptoms, which lasted up until one month postoperatively and resolved following oral calcium and vitamin D supplementation. No patient developed permanent hypocalcemia.

The univariate analysis did not identify age or gender as risk factors for IPT (Table 3). Similarly, thyroid gland weight, the diagnosis of malignancy, or the presence of Hashimoto thyroiditis were not found to correlate with IPT.

Biochemical hypocalcemia was noticed in 28.6% (20/70) of cases in IPT group and in 13.3% (28/211) in the non-IPT group (*p* = 0.003). On the contrary, symptomatic hypocalcemia was observed in 5.7% (4/70) in the IPT group and in 3.8% (8/211) in the non-IPT group (*p* = 0.49).

While preoperative values were similar, both calcium and phosphate levels differed significantly in the postoperative setting (Table 4), with significantly lower calcium (*p* = 0.0008) and higher phosphate levels (*p* = 0.007) in those patients with 2 or more PGs accidentally removed.

## 4. Discussion

Parathyroid glands were first described by Ivar Sandstrom in 1880, but it was not until 1908 that MacCallum and Voegtlin investigated their pivotal role in calcium metabolism [5].

The two upper PGs derive their origin from the fourth pharyngeal pouch and descent along with the thyroid, while the two lower PGs develop from the third pharyngeal pouch with the thymus and follow its downward migration towards the anterior mediastinum [6]. The position of the upper PGs is fairly consistent, with the cricothyroid junction and upper pole of thyroid being the most common sites. The lower PGs are more frequently ectopic and with wider distribution (lower thyroid pole, thyrothymic ligament, and upper and lower mediastinum) [7].

Supernumerary PGs are discovered in about 2–6% of patients [7, 8]. On the contrary, fewer than four PGs are reported in 3% of cases, but this may be partly attributed to failure of recognition of an ectopic fourth PG during dissection, rather than true agenesis of the fourth gland [7, 8]. A large cadaveric study in a Greek population revealed 4 PGs in 93% of cases, with the vast majority of them (>90%) being orthotopic [7]. The authors also found a notably low prevalence of intrathyroidal PGs (0.2% of all PGs, 2% of ectopic PGs).

This variability in location, number, size, and shape renders them vulnerable to iatrogenic trauma. The incidence of IPT in our series was 24.9%, which is at the upper quartile of the rates reported in the international literature (Table 5). Interestingly, we found a relatively high percentage of intrathyroidal PGs (44.3%) compared to previously published data (Table 5). Two older studies from Greece revealed a similar prevalence of intrathyroidal PGs (42.6 and 49%), while Sakorafas and Spiliotis reported rates of 21 and 28%, respectively [4, 9–11].

In theory, careful examination of the thyroid gland during surgery and immediately after resection may allow for recognition of extra- and subcapsular PGs, which in turn may be autotransplanted after frozen section confirmation [6]. On the contrary, intrathyroidal PGs may lie in fissures of the thyroid surface or be truly intrathyroidal, practically precluding positive identification, even with a meticulous technique close to the thyroid capsule [7, 12].

However the value of systemic identification of all four PGs remains as a matter of controversy. Recent studies suggest that identifying a greater number of PGs in situ does not necessarily lead to a reduction in the postoperative rates of hypocalcemia [12, 25]. The authors hypothesized that extensive dissection for missing PGs may cause devascularization or direct injury and may be required only in cases of central neck dissection [17, 25].

On the other hand, rates of permanent hypocalcemia increase when fewer than two to three PGs are preserved [26, 27]. This would justify autotransplantation of accidentally extirpated PGs [6, 28, 29]. In the present series, more than half of IPTs could theoretically have been avoided, since the PGs were found in extra- and subcapsular locations. However, when adequate parathyroid mass has been left behind, permanent hypocalcemia will rarely develop [3, 17].

Previous studies have variably identified younger age [14, 19, 23], female sex [4, 9, 14], Hashimoto thyroiditis [18, 22], and malignancy [13, 15, 16, 19, 23, 30] as potential risk factors for IPT. Of notice, Sakorafas and Gourgiotis reported lower rates of IPT in patients with malignancy, owing probably to more meticulous dissection in cases of suspected malignancy [4, 10]. Our analysis did not reveal any of the aforementioned factors to affect the rates of IPT. In addition, more extensive procedures have been traditionally associated with higher risk for IPT, due to radicality (neck lymph node dissections) or abutment of the normal anatomical planes by scarring (reoperations) [16–24]. In this series, patients undergoing completion thyroidectomy and cervical lymphadenectomy were excluded by the study protocol.

On the other hand, we found that IPT in our patients was associated with transient biochemical hypocalcemia, but not with symptomatic disease. Evidence in the literature linking IPT to postoperative hypocalcemia and serum calcium levels remains controversial (Table 5) and may depend on the actual definitions of hypocalcemia and parathyroid failure [1, 31].

The number of in situ preserved PGs seems to play a role in the postoperative calcium/phosphate homeostasis. In our series, serum calcium and phosphate levels did not differ significantly between patients with none or only one PG removed. However this difference did reach statistical significance in the subgroup of patients with 2 or more PGs extirpated. Even if permanent hypocalcemia can be prevented by the preservation of a single viable PG, parathyroid autotransplantation appears as a reasonable decision in this subgroup of patients [16, 17].

Limitations of our study protocol include its single-institution, retrospective nature and relatively small sample size. The study population however was homogenous in terms of the surgical intervention performed, the juxtacapsular dissection technique, and the single use of hemostatic technique. On the other hand, routine postoperative PTH assessment was implemented in our department only in 2010; therefore we chose not to include it in the present analysis. Finally, the meticulous examination of the thyroid specimen and the thin 2 mm sections allowed for precise identification of parathyroid tissue, which otherwise might have been overlooked.

## 5. Conclusion

Incidental parathyroidectomy is not uncommon following thyroid surgery, even in the hands of experienced surgeons. In this 10-year audit of total thyroidectomies, incidence of IPT reached 24.9%, with 44.3% of those PGs found in an intrathyroidal location, virtually precluding autotransplantation. Age, sex, thyroid specimen weight, Hashimoto thyroiditis, and malignancy were not recognized as risk factors. Incidental parathyroidectomy was associated with transient biochemical hypocalcemia, but not with clinically symptomatic disease. Patients with 2 or more PGs resected had significantly lower calcium and higher phosphate levels postoperatively, and in those cases PG autotransplantation may be beneficial.

## Figures and Tables

**Table 1:** Demographic and pathological characteristics.

	*n*	%
*Sex*		
Male	63	22.4%
Female	218	77.6%
*Age*	54.3 ± 14.4 years	Range 18–85 years
*Thyroid gland weight*	34.9 ± 29.1 gr	Range 5–212 gr
*Thyroid pathology*		
Benign	195	69.4%
Malignant	86	30.6%
*Diagnosis*		
Multinodular goiter	150	53.4%
Graves	8	2.8%
Hashimoto	30	10.7%
Follicular adenoma	7	2.5%
Papillary thyroid cancer	79	28.1%
Follicular carcinoma	3	1.1%
Myeloid thyroid cancer	3	1.1%
Anaplastic thyroid cancer	1	0.3%

**Table 2:** Incidental parathyroidectomy.

	*n*	%
*Incidental parathyroidectomy*	70	24.9%
*Number of removed PGs*		
1	48	68.6%
2	14	20.0%
3	3	4.3%
Fragment	5	7.1%
*Autotransplantation of PGs*	4	5.7%
*Mean diameter of removed PGs*	4.1 mm	
*Location of removed PGs*		
Intrathyroidal	31	44.3%
Subcapsular	28	40.0%
Extracapsular	11	15.7%
*Histology of removed PGs*		
Normal	66	94.3%
Hyperplasia without atypia	4	5.7%
Adenoma	0	0

**Table 3:** Comparison of incidental parathyroidectomy (IPT) and nonincidental parathyroidectomy (non-IPT) groups.

	IPT group	Non-IPT group	*p* value
*N*	70	211	
*Age (years)*	55.4 ± 14.7	54.0 ± 14.4	0.24
*Sex*			
Male	15 (21.4%)	48 (22.7%)	
Female	55 (78.6%)	163 (77.3)	0.82
*Thyroid gland weight (gr)*	36.3 ± 26.1	34.4 ± 30.1	0.31
*Diagnosis*			
Benign	47 (67.1%)	148 (70.1%)	
Malignant	23 (32.9%)	63 (29.9%)	0.63
*Hashimoto thyroiditis*			
Yes	9 (12.8%)	33 (15.6%)	
No	61 (87.2)	178 (84.4%)	0.57
*Biochemical hypocalcemia*			
Yes	20 (28.6%)	28 (13.3%)	
No	50 (71.4%)	183 (86.7%)	**0.003**
*Clinical hypocalcemia*			
Yes	4 (5.7%)	8 (3.8%)	
No	66 (94.3%)	203 (96.2%)	0.49

**Table 4:** Pre- and postoperative calcium (Ca^++^) and phosphate (Ph) levels.

	No PGs resected	One PG resected	≥2 PGs resected	*p* value
Pre-op Ca^++^	9.31 ± 0.53	9.49 ± 0.57	9.37 ± 0.56	0.18
Post-op Ca^++^	8.65 ± 0.71	8.42 ± 0.69	7.98 ± 0.57	**0.0008**
Pre-op Ph	3.45 ± 0.53	3.55 ± 0.56	3.55 ± 0.60	0.47
Post-op Ph	3.88 ± 0.74	3.96 ± 0.78	4.52 ± 0.84	**0.007**

**Table 5:** Previous studies on incidental parathyroidectomy (IPT).

Name	*n*	IPT (%)	Intrathyroidal PGs%	Conclusion
Özoğul et al. [13]	801	2.3	5.2	*IPT associated with hypocalcemia*
Praženica et al. [14]	788	6.6	20.3	*IPT associated with temporary and permanent hypocalcemia*
Qasaimeh et al. [15]	233	8.6	21.7	*IPT associated with transient hypocalcemia*
Youssef et al. [16]	207	12.6	57.7	IPT not associated with hypocalcemia
Song et al. [17]	454	19.8	—	*IPT associated with transient hypocalcemia*
Erbil et al. [18]	440	10.9	68.8	IPT not associated with hypocalcemia
Manouras et al. [9]	508	19.7	49	IPT not associated with hypocalcemia
Sippel et al. [19]	513	6.4	—	*IPT associated with biochemical hypocalcemia*
Gourgiotis et al. [10]	315	21.6	42.6	IPT not associated with hypocalcemia
Sakorafas et al. [4]	158	17.7	21	IPT not associated with hypocalcemia
Sasson et al. [20]	141	15	50	IPT not associated with hypocalcemia
Lin et al. [21]	220	9.1	15	IPT not associated with hypocalcemia
Khairy and Al-Saif [22]	287	16.4	42.9	*IPT associated with hypocalcemia*
Sorgato et al. [23]	882	7.9	15.7	IPT not associated with hypocalcemia
Abboud et al. [6]	307	12	24	IPT not associated with hypocalcemia
Rix and Sinha [24]	126	17.4	13.6	IPT not associated with hypocalcemia
Sheahan et al. [25]	126	9.5	—	IPT not associated with hypocalcemia
Spiliotis et al. [11]	315	10.2	28.1	*IPT associated with hypocalcemia*
